# Supplementary material for: End Sequence Analysis Toolkit (ESAT) expands the extractable information from single-cell RNA-seq data
Source: Genome Res. 2016 Oct;26(10):1397–410. doi: 10.1101/gr.207902.116 (PMC5052061; doi:10.1101/gr.207902.116)
Supplement: Supplemental Material [file supp_gr.207902.116_Supp_Materials.tar.gz › ESAT-esat_v0.1/doc/html/classumms_1_1core_1_1alignment_1_1_feature_sequence_alignment.html]

ESAT: umms.core.alignment.FeatureSequenceAlignment Class Reference


ESAT


- Main Page
- Related Pages
- Packages
- Classes
- Files

- Class List
- Class Index
- Class Hierarchy
- Class Members

All Classes Namespaces Files Functions Variables Enumerator Pages

- umms
- core
- alignment
- FeatureSequenceAlignment

Classes |
Public Member Functions |
Static Public Member Functions |
Static Public Attributes |
List of all members

umms.core.alignment.FeatureSequenceAlignment Class Reference

|  |  |
| --- | --- |
| Classes | |
| class | UnorderedGenePair |
|  | |

|  |  |
| --- | --- |
| Public Member Functions | |
|  | FeatureSequenceAlignment (String featureBedFile, String genomeFasta) throws IOException |
|  | |
|  | FeatureSequenceAlignment (Collection< Gene > genes, String genomeFasta) throws IOException |
|  | |
|  | FeatureSequenceAlignment (String featureBedFile, String genomeFasta, float matchScore, float mismatchScore, float gapOpenPenalty, float gapExtendPenalty) throws IOException |
|  | |
|  | FeatureSequenceAlignment (Collection< Gene > genes, Map< String, broad.core.sequence.Sequence > chrs, float matchScore, float mismatchScore, float gapOpenPenalty, float gapExtendPenalty) throws IOException |
|  | |
|  | FeatureSequenceAlignment (Collection< Gene > genes, String genomeFasta, float matchScore, float mismatchScore, float gapOpenPenalty, float gapExtendPenalty) throws IOException |
|  | |
| Map< UnorderedGenePair,   jaligner.Alignment > | getAllPairwiseSenseAlignments (float minAlignLength, float minPctIdentity) |
|  | |
| Map< UnorderedGenePair,   jaligner.Alignment > | getAllPairwiseAntisenseAlignments (float minAlignLength, float minPctIdentity) |
|  | |
| void | writeAllSenseAlignmentsToBed (String outBedFile, int minAlignLength, float minPctIdentity) throws IOException |
|  | |
| void | writeAllAntisenseAlignmentsToBed (String outBedFile, int minAlignLength, float minPctIdentity) throws IOException |
|  | |
| void | writeAllSenseAlignmentsToBed (String outBedFile, boolean append, int minAlignLength, float minPctIdentity) throws IOException |
|  | |
| void | writeAllAntisenseAlignmentsToBed (String outBedFile, boolean append, int minAlignLength, float minPctIdentity) throws IOException |
|  | |

|  |  |
| --- | --- |
| Static Public Member Functions | |
| static void | main (String[] args) throws IOException |
|  | |

|  |  |
| --- | --- |
| Static Public Attributes | |
| static float | DEFAULT\_MATCH\_SCORE = 5 |
|  | |
| static float | DEFAULT\_MISMATCH\_SCORE = -4 |
|  | |
| static float | DEFAULT\_GAP\_OPEN\_PENALTY = 8 |
|  | |
| static float | DEFAULT\_GAP\_EXTEND\_PENALTY = 2 |
|  | |

## Detailed Description

Author
:   prussell

## Constructor & Destructor Documentation

|  |  |  |  |
| --- | --- | --- | --- |
| umms.core.alignment.FeatureSequenceAlignment.FeatureSequenceAlignment | ( | String | *featureBedFile*, |
|  |  | String | *genomeFasta* |
|  | ) |  | throws IOException |

Parameters
:   |  |  |
    | --- | --- |
    | featureBedFile | Bed file of features |
    | genomeFasta | Genome fasta file |

Exceptions
:   |  |  |
    | --- | --- |
    | IOException |  |

Here is the caller graph for this function:

|  |  |  |  |
| --- | --- | --- | --- |
| umms.core.alignment.FeatureSequenceAlignment.FeatureSequenceAlignment | ( | Collection< Gene > | *genes*, |
|  |  | String | *genomeFasta* |
|  | ) |  | throws IOException |

Parameters
:   |  |  |
    | --- | --- |
    | genes | Set of features |
    | genomeFasta | Genome fasta file |

Exceptions
:   |  |  |
    | --- | --- |
    | IOException |  |

|  |  |  |  |
| --- | --- | --- | --- |
| umms.core.alignment.FeatureSequenceAlignment.FeatureSequenceAlignment | ( | String | *featureBedFile*, |
|  |  | String | *genomeFasta*, |
|  |  | float | *matchScore*, |
|  |  | float | *mismatchScore*, |
|  |  | float | *gapOpenPenalty*, |
|  |  | float | *gapExtendPenalty* |
|  | ) |  | throws IOException |

Parameters
:   |  |  |
    | --- | --- |
    | featureBedFile | Bed file of features |
    | genomeFasta | Genome fasta file |
    | matchScore | Match score for Smith Waterman |
    | mismatchScore | Mismatch score for Smith Waterman |
    | gapOpenPenalty | Gap open penalty for Smith Waterman |
    | gapExtendPenalty | Gap extend penalty for Smith Waterman |

Exceptions
:   |  |  |
    | --- | --- |
    | IOException |  |

|  |  |  |  |
| --- | --- | --- | --- |
| umms.core.alignment.FeatureSequenceAlignment.FeatureSequenceAlignment | ( | Collection< Gene > | *genes*, |
|  |  | Map< String, broad.core.sequence.Sequence > | *chrs*, |
|  |  | float | *matchScore*, |
|  |  | float | *mismatchScore*, |
|  |  | float | *gapOpenPenalty*, |
|  |  | float | *gapExtendPenalty* |
|  | ) |  | throws IOException |

Parameters
:   |  |  |
    | --- | --- |
    | genes | The features |
    | chrs | Map of chromosome names to sequences |
    | matchScore | Match score for Smith Waterman |
    | mismatchScore | Mismatch score for Smith Waterman |
    | gapOpenPenalty | Gap open penalty for Smith Waterman |
    | gapExtendPenalty | Gap extend penalty for Smith Waterman |

Exceptions
:   |  |  |
    | --- | --- |
    | IOException |  |

|  |  |  |  |
| --- | --- | --- | --- |
| umms.core.alignment.FeatureSequenceAlignment.FeatureSequenceAlignment | ( | Collection< Gene > | *genes*, |
|  |  | String | *genomeFasta*, |
|  |  | float | *matchScore*, |
|  |  | float | *mismatchScore*, |
|  |  | float | *gapOpenPenalty*, |
|  |  | float | *gapExtendPenalty* |
|  | ) |  | throws IOException |

Parameters
:   |  |  |
    | --- | --- |
    | genes | Set of features |
    | genomeFasta | Genome fasta file |
    | matchScore | Match score for Smith Waterman |
    | mismatchScore | Mismatch score for Smith Waterman |
    | gapOpenPenalty | Gap open penalty for Smith Waterman |
    | gapExtendPenalty | Gap extend penalty for Smith Waterman |

Exceptions
:   |  |  |
    | --- | --- |
    | IOException |  |

## Member Function Documentation

|  |  |  |  |
| --- | --- | --- | --- |
| Map<UnorderedGenePair, jaligner.Alignment> umms.core.alignment.FeatureSequenceAlignment.getAllPairwiseAntisenseAlignments | ( | float | *minAlignLength*, |
|  |  | float | *minPctIdentity* |
|  | ) |  |  |

Get all antisense direction alignments passing thresholds

Parameters
:   |  |  |
    | --- | --- |
    | minAlignLength | Min alignment length |
    | minPctIdentity | Min percent identity |

Returns
:   All antisense alignments of unordered gene pair passing criteria

Here is the caller graph for this function:

|  |  |  |  |
| --- | --- | --- | --- |
| Map<UnorderedGenePair, jaligner.Alignment> umms.core.alignment.FeatureSequenceAlignment.getAllPairwiseSenseAlignments | ( | float | *minAlignLength*, |
|  |  | float | *minPctIdentity* |
|  | ) |  |  |

Get all sense direction alignments passing thresholds

Parameters
:   |  |  |
    | --- | --- |
    | minAlignLength | Min alignment length |
    | minPctIdentity | Min percent identity |

Returns
:   All sense alignments of unordered gene pair passing criteria

Here is the caller graph for this function:

|  |  |  |  |  |  |
| --- | --- | --- | --- | --- | --- |
| static void umms.core.alignment.FeatureSequenceAlignment.main | ( | String[] | *args* | ) | throws IOException |

static

Parameters
:   |  |  |
    | --- | --- |
    | args |  |

Exceptions
:   |  |  |
    | --- | --- |
    | IOException |  |

Here is the call graph for this function:

|  |  |  |  |
| --- | --- | --- | --- |
| void umms.core.alignment.FeatureSequenceAlignment.writeAllAntisenseAlignmentsToBed | ( | String | *outBedFile*, |
|  |  | int | *minAlignLength*, |
|  |  | float | *minPctIdentity* |
|  | ) |  | throws IOException |

Write all pairwise alignments (antisense direction only) to bed file in genome coordinates

Parameters
:   |  |  |
    | --- | --- |
    | outBedFile | Output bed file |
    | minAlignLength | Min alignment length to keep |
    | minPctIdentity | Min percent identity to keep |

Exceptions
:   |  |  |
    | --- | --- |
    | IOException |  |

|  |  |  |  |
| --- | --- | --- | --- |
| void umms.core.alignment.FeatureSequenceAlignment.writeAllAntisenseAlignmentsToBed | ( | String | *outBedFile*, |
|  |  | boolean | *append*, |
|  |  | int | *minAlignLength*, |
|  |  | float | *minPctIdentity* |
|  | ) |  | throws IOException |

Write all pairwise alignments (antisense direction only) to bed file in genome coordinates

Parameters
:   |  |  |
    | --- | --- |
    | outBedFile | Output bed file |
    | append | Write to end of file rather than beginning |
    | minAlignLength | Min alignment length to keep |
    | minPctIdentity | Min percent identity to keep |

Exceptions
:   |  |  |
    | --- | --- |
    | IOException |  |

Here is the call graph for this function:

|  |  |  |  |
| --- | --- | --- | --- |
| void umms.core.alignment.FeatureSequenceAlignment.writeAllSenseAlignmentsToBed | ( | String | *outBedFile*, |
|  |  | int | *minAlignLength*, |
|  |  | float | *minPctIdentity* |
|  | ) |  | throws IOException |

Write all pairwise alignments (sense direction only) to bed file in genome coordinates

Parameters
:   |  |  |
    | --- | --- |
    | outBedFile | Output bed file |
    | minAlignLength | Min alignment length to keep |
    | minPctIdentity | Min percent identity to keep |

Exceptions
:   |  |  |
    | --- | --- |
    | IOException |  |

|  |  |  |  |
| --- | --- | --- | --- |
| void umms.core.alignment.FeatureSequenceAlignment.writeAllSenseAlignmentsToBed | ( | String | *outBedFile*, |
|  |  | boolean | *append*, |
|  |  | int | *minAlignLength*, |
|  |  | float | *minPctIdentity* |
|  | ) |  | throws IOException |

Write all pairwise alignments (sense direction only) to bed file in genome coordinates

Parameters
:   |  |  |
    | --- | --- |
    | outBedFile | Output bed file |
    | append | Write to end of file rather than beginning |
    | minAlignLength | Min alignment length to keep |
    | minPctIdentity | Min percent identity to keep |

Exceptions
:   |  |  |
    | --- | --- |
    | IOException |  |

Here is the call graph for this function:

## Member Data Documentation

float umms.core.alignment.FeatureSequenceAlignment.DEFAULT\_GAP\_EXTEND\_PENALTY = 2

static

Default gap extend penalty for Smith Waterman

float umms.core.alignment.FeatureSequenceAlignment.DEFAULT\_GAP\_OPEN\_PENALTY = 8

static

Default gap open penalty for Smith Waterman

float umms.core.alignment.FeatureSequenceAlignment.DEFAULT\_MATCH\_SCORE = 5

static

Default match score for Smith Waterman

float umms.core.alignment.FeatureSequenceAlignment.DEFAULT\_MISMATCH\_SCORE = -4

static

Default mismatch score for Smith Waterman

---

The documentation for this class was generated from the following file:

- java/umms/core/alignment/FeatureSequenceAlignment.java


---

Generated on Fri Aug 15 2014 15:11:07 for ESAT by  

 1.8.7
